# Auxin Signaling in Regulation of Plant Translation Reinitiation

**DOI:** 10.3389/fpls.2017.01014

**Published:** 2017-06-14

**Authors:** Mikhail Schepetilnikov, Lyubov A. Ryabova

**Affiliations:** Institut de Biologie Moléculaire des Plantes, Centre National de la Recherche Scientifique, UPR 2357, Université de StrasbourgStrasbourg, France

**Keywords:** target of rapamycin TOR, small GTPases ROPs, S6K1, endosomes, signal transduction, translation-reinitiation

## Abstract

The mRNA translation machinery directs protein production, and thus cell growth, according to prevailing cellular and environmental conditions. The target of rapamycin (TOR) signaling pathway—a major growth-related pathway—plays a pivotal role in optimizing protein synthesis in mammals, while its deregulation triggers uncontrolled cell proliferation and the development of severe diseases. In plants, several signaling pathways sensitive to environmental changes, hormones, and pathogens have been implicated in post-transcriptional control, and thus far phytohormones have attracted most attention as TOR upstream regulators in plants. Recent data have suggested that the coordinated actions of the phytohormone auxin, Rho-like small GTPases (ROPs) from plants, and TOR signaling contribute to translation regulation of mRNAs that harbor upstream open reading frames (uORFs) within their 5′-untranslated regions (5′-UTRs). This review will summarize recent advances in translational regulation of a specific set of uORF-containing mRNAs that encode regulatory proteins—transcription factors, protein kinases and other cellular controllers—and how their control can impact plant growth and development.

## Introduction

Plant hormones (phytohormones) trigger complex growth and developmental processes. One of the most important plant growth regulators is auxin (from the Greek “auxein” meaning to enlarge/grow)—a small signaling molecule with great ability to induce growth responses throughout the plant life cycle. The auxin signaling pathway modulates diverse aspects of plant growth and development, such as responses to light and gravity, organ patterning, general root and shoot architecture and vascular development. Auxin elicits responses—cell division and expansion—depending on the cellular and developmental context in which it is perceived. The core components of auxin signaling differ in their expression patterns due to transcriptional and post-transcriptional regulation. Here, we review recent data describing auxin signaling in the cytoplasm of plant cells, and how auxin perception leads to activation of target of rapamycin (TOR), which promotes a protein synthesis pathway. In eukaryotes, TOR signaling is a key signaling pathway connecting environmental signal perception to growth decisions. Thus, TOR is a sensor that up-regulates cell growth and proliferation but also limits life span in yeast, mammals and plants. A hypothetical scheme linking auxin and TOR signaling with the G-protein (guanine nucleotide-binding proteins) family is described. This observation makes TOR an important part of the auxin signaling pathway that up-regulates translation, and, thus, plant growth and development.

It is clear that many environmental cues, such as nutrient and energy availability, instruct phytohormones to control plant growth, making it a very plastic process. We decided to travel along the recently discovered pathway from auxin to TOR via a small GTPase, ROP2, which ends by up-regulating production of critical effector proteins using a post-transcriptional mechanism via targeting of a specific translation initiation pathway: reinitiation.

## Auxin Perception and Signaling

Auxin distribution is highly regulated in plants. Local auxin maxima and concentration gradients drive cell differentiation and embryogenesis. Auxin patterns form dynamically in response to environmental inputs (e.g., light and gravity). Thus auxin signal is converted into context-dependent developmental responses. Auxin perception is believed to be mediated by receptors that physically bind auxin, allowing it to travel from outside the cell into the cell cytoplasm, where it then initiates signal transduction cascades that trigger specific physiological auxin responses.

The best-characterized auxin pathway targets the nucleus (Mockaitis and Estelle, 2008), whereas the cytoplasmic role of auxin remains unexplored despite the existence of cytoplasmic auxin networks in the ER and plasma membranes (PMs) (Friml and Jones, 2010). The classical nuclear auxin signaling pathway relies on a molecular mechanism of action via auxin-dependent degradation of the transcriptional repressors Aux/IAA, which leads to gene activation outputs depending on the cellular spatio-temporal context. This degradation is dependent on the ubiquitin ligase Skp1-Cullin-F-box (SCF)^TIR1^ protein complex, where the associated F-box protein TIR1 confers target specificity (Lavy and Estelle, 2016). In the presence of auxin, the F-box protein TIR1 binds to Aux/IAA, resulting in the ubiquitination and degradation of the latter (Gray et al., 2001). By filling in a hydrophobic cavity at the protein interface, auxin enhances TIR1–substrate interactions by acting as a “molecular glue” (Tan et al., 2007). In this context, F-box protein TIR1 is a true auxin receptor, mediating transcriptional responses to auxin in plants (Dharmasiri et al., 2005; Kepinski and Leyser, 2005). Each TIR receptor targets specific Aux/IAA proteins for degradation (Parry et al., 2009), thus switching on transcription of a multitude of genes, including auxin response factors (ARFs). The ARF transcription factors (23 members in *Arabidopsis*) contain DNA binding domains and interact specifically with tandem repeats, known as *Auxin-Responsive Elements* (*AuxREs*; Ulmasov et al., 1995, 1997a,b) that serve as either activators or repressors of transcription. ARFs regulate a multitude of critical steps in plant development by converting local auxin maxima into gene expression responses. Several *ARF* genes confer developmental phenotypes, and some possess interaction complexity and functional overlap. One example is ETTIN/ARF3, which is involved in establishment of organ polarity (Sessions et al., 1997; Garcia et al., 2006; Marin et al., 2010; Kelley et al., 2012). Recent data suggest the existence of a non-canonical direct auxin effect on ETTIN without the ubiquitination and Aux/IAA-mediated degradation steps (Simonini et al., 2016), thus raising the question of whether alternative auxin pathways can exist. Another well-studied example of a transcription-activating ARF is MONOPTEROS/ARF5 (MP). Defects in MP result in aberrant seedling morphology, often with a single cotyledon and a loss of basal structures (Hardtke and Berleth, 1998). Current data indicate that ARF protein levels are regulated post-transcriptionally (Nishimura et al., 2005; Leyser, 2006; Zhou et al., 2010).

Auxin Binding Protein 1 (ABP1), which displays high affinity to chlorinated auxins (Reinard et al., 1998; Napier et al., 2002), was characterized as an auxin receptor and implicated in many aspects of growth and development, particularly mediating the fast, non-genomic effects of auxin (for a review, see Chen and Yang, 2014). Specifically, ABP1 was implicated in rapid cell surface-located auxin signaling as a sensor of cytosolic pH and K^+^ flux (Thiel et al., 1993; Gehring et al., 1998). Although ABP1 is a soluble auxin receptor, its partnering with membrane associated-receptor-like kinase TMK (transmembrane kinase) was proposed for perception of auxin and its travel to the cytoplasm (Xu et al., 2014). In 2015, however, several publications raised significant concerns about the role of ABP1 in both auxin signaling and *Arabidopsis* development (Gao et al., 2015; Strader and Zhao, 2016).

Since auxin efflux carriers bind auxin and promote its polar active transport (PAT) from cell to cell, it was suggested that the PIN-FORMED (PIN) family of auxin efflux carriers could be considered as auxin receptors (Hertel, 1995). PINs orchestrate polar cell-to-cell auxin transport via asymmetric subcellular concentrations. Moreover, PINs were implicated in the formation of auxin perception complexes when partnered with PID (PINOID) protein kinases (for a review, see Strader and Zhao, 2016). However, since PINs are not able to generate secondary messengers or the intermediate reactions required for signal transduction, this idea seemed to be non-productive. Interestingly, several PINs, including PIN5 and PIN8, are involved in cytoplasmic auxin trafficking, where PIN5 likely mediates auxin transport from the cytosol into the lumen of the ER (Mravec et al., 2009), and PIN8 from the ER to the cytosol (Ding et al., 2012). PIN8 is highly expressed during pollen development, and resides in the ER of pollen grains and germinated tubes (Bosco et al., 2012; Ding et al., 2012). Although PIN8 specific expression resulted in shorter root hairs likely due to auxin efflux activities that decrease accumulation of auxin, overexpression of PIN5 promotes root hair growth by increasing levels of internal auxin in the root hair cells (Ganguly et al., 2010). Therefore, both PIN5 and PIN8 can mediate auxin trafficking within the cytosol and the ER, but their output effects require further studies.

Auxin can alter plant development rapidly in response to different environmental stimuli acting at many diverse downstream target systems. In the cytoplasm, auxin is able to activate PM-associated ROPs (Rho-like GTPases from plants), which are involved in the regulation of endocytosis of auxin transport proteins and organization of the cytoskeleton (Tao et al., 2002). Although ROPs, as powerful signaling molecules, coordinate many diverse signal transduction pathways, accumulating data suggest clearly defined crosstalk between auxin and ROP signaling (Tao et al., 2002; Xu et al., 2010; Schepetilnikov et al., 2017). ROP GTPases function as mediators of auxin-regulated gene expression, rapid PM auxin signaling, and directional auxin transport to link local auxin gradients with ROP regulation of cell polarity (for a review, see Wu et al., 2011). In *Arabidopsis*, ROPs are encoded by 11 genes that comprise a closely related, multigenic family that represents a subgroup of the Ras superfamily of small GTPases, and includes Rho, Rac, and Cdc42 subfamilies (Winge et al., 2000). Like other G-proteins, ROPs interact with their target proteins through conformation-specific states: a GTP-bound active state, a short-lived nucleotide free state, and a GDP-bound inactive state (Berken and Wittinghofer, 2008). ROPs efficiently bind GTP, but their hydrolysis activity depends on Rho GTPase-activating proteins RopGAPs (Berken et al., 2005; Gu et al., 2006; Berken and Wittinghofer, 2008). Plants contain a family of RhoGAPs that carries a conserved GAP-related domain and an N-terminal CRIB (Cdc42/Rac-interactive binding) motif that is involved in ROP binding (Wu et al., 2000), and REN1 (ROP1 ENHANCER1) protein, which, in addition to a GAP-related domain, carries an N-terminal pleckstrin homology (PH) domain (Hwang et al., 2008). Both RopGAPs have been shown to regulate ROP signaling (Wu et al., 2000; Klahre and Kost, 2006; Hwang et al., 2008). In contrast, guanine nucleotide exchange factors (RopGEFs) activate ROPs by promoting GDP–GTP recycling. The *Arabidopsis* genome contains a single ortholog of the mammalian DOC180 family protein, SPIKE1 (SPK1; Qiu et al., 2002), and 14 plant-specific RopGEF family members with the PRONE (plant-specific Rop nucleotide exchanger) domain required for GTP–GDP exchange (Berken et al., 2005; Gu et al., 2006). Several ROP downstream effectors—a family of CRIB-domain-containing proteins (RICs) that specifically interact with active GTP-bound ROPs—have been described in plants (Nagawa et al., 2010; Fehér and Lajkó, 2015). Although RICs are highly variable, their CRIB motifs are highly conserved (Nagawa et al., 2010), and the CRIB motif is used widely to estimate active ROP levels using a pull-down assay with a ROP-interactive CRIB motif-containing protein 1 (Ric1) that specifically targets activated forms of RAC/ROPs (Tao et al., 2002).

Although the role of the auxin signal transduction pathway in ROP signaling activation has been well documented, mechanisms and intermediate signaling components are not well known. Since RopGEFs are defined as molecules that activate ROPs, while RopGAPs prevents uncontrolled signaling of ROPs, both could be potential components of the auxin-ROP signaling axis. The PM localized receptor-like protein kinases (RLKs) play a critical role in transmission of extracellular signals to intracellular ROP signaling pathways, and function in regulation of fertilization and cell expansion mechanisms such as cell elongation, tip and hair growth (for a recent review, see Galindo-Trigo et al., 2016). We draw the reader’s attention to the *Catharanthus roseus* RLK-1-like (CrRLK1L) protein kinase subfamily, which contains FERONIA (FER; Hématy and Höfte, 2008). FER specifically up-regulates ROP2 signaling activity through RopGEFs in *Arabidopsis* (Duan et al., 2010); the FER and RopGEF-containing complex recruits an inactive form of ROP2 and converts it to an active form in a guanine nucleotide-responsive manner, while *fer* mutants accumulate the inactive (GDP) form of ROP2 (Duan et al., 2010). Moreover, it was suggested that a network of different RLKs, RhoGEFs, and ROPs can respond to diverse signals in various tissue and cell types (Schiller, 2006). Importantly, FER protein kinase interferes with several phytohormone pathways, including auxin signaling (Duan et al., 2010). Although auxin signaling stimulates root hair elongation (Pitts et al., 1998; Rahman et al., 2002), root hairs of *fer* mutants are not responsive to exogenous application of auxin (Duan et al., 2010). Taking into account that loss-of-function *fer* mutants are pleiotropic and display severe growth defects, FER is indispensable for plant growth and development (Duan et al., 2010). Future research will determine whether FER and RopGEFs function in ROP signaling control in an auxin-sensitive manner. Generally, RLKs can have broad functions in regulating cytoskeletal organization, vesicle trafficking and reactive oxygen species (ROS) production during plant growth (Wolf and Höfte, 2014).

## TOR Signaling Complexes and their Upstream Regulation

Cell growth requires protein synthesis—a process that consumes a huge amount of energy and therefore needs to be tightly regulated to keep a balance between cell demands and resources. Plants and animals share a common signaling pathway—the TOR pathway—connecting growth with environmental signal perception, where TOR accomplishes fine-tuning of the translational machinery, thus reprogramming translation rates in accordance with cellular needs. TOR operates as a hub in the signal transduction network that coordinates many critical molecular processes in eukaryotes, such as translation, proliferation, transcription, survival, aging, differentiation and autophagy, and is responsive to diverse signals, including nutrient and oxygen availability, energy sufficiency, stress, hormones, and growth factors. For two recent excellent reviews on the TOR signaling pathway in plants (see Barrada et al., 2015; Dobrenel et al., 2016).

Target of rapamycin belongs to the family of phosphatidylinositol kinase-related kinases (PIKKs), and is clearly related to PIK. However, TOR is atypical of PIK in that it appears not to phosphorylate lipid substrates, instead possessing a serine–threonine protein kinase activity. TOR was first described in yeast over 20 years ago as a target protein of the anti-fungal and immunosuppressant agent rapamycin (Heitman et al., 1991; Kunz et al., 1993). Rapamycin is a naturally occurring macrolide that acts as an allosteric inhibitor of TOR. Rapamycin forms a drug–receptor complex with the cellular peptidyl-prolyl *cis-trans* isomerase FKBP12, which, upon binding to TOR, inhibits its kinase activity (Sabatini et al., 1994; Choi et al., 1996). In contrast, most plants are insensitive to rapamycin-mediated inhibition of growth due to FKBP12, which is not efficient in rapamycin binding (Xu et al., 1998; Mahfouz et al., 2006; Sormani et al., 2007; Deng et al., 2016). Mammalian TOR (mTOR) exists in two multiprotein complexes, mTORC1 and mTORC2, which differ in their composition, function, downstream substrates, and mode of action (direct or indirect) in many cellular processes. mTORC1 contains the TOR catalytic subunit, scaffold protein Raptor (regulatory associated protein of TOR), adaptor mLst8 (lethal with SEC13 protein 8) and regulatory protein DEPTOR (DEP domain-containing TOR-interacting). The mTORC2 complex—larger in size, with a molecular weight of about 1.4 MDa—contains TOR, scaffold protein Rictor (rapamycin-insensitive companion of TOR), mLst8, hSin1 (stress-activated protein kinase-interacting protein 1), PROTOR (protein observed with Rictor) and DEPTOR. mTORC1 is typically defined by a specific component, Raptor, and stimulates anabolic processes, including protein synthesis (Ma and Blenis, 2009), whereas mTORC2 contains Rictor and regulates cytoskeletal organization and survival (Hara et al., 2002; Kim et al., 2002; Loewith et al., 2002). mTORC2 is activated by the ribosome, where TORC2-ribosome interaction is a likely conserved mechanism that is physiologically relevant in both normal and cancer cells (Zinzalla et al., 2011). In addition, under most conditions, mTORC1 is sensitive to rapamycin, but mTORC2 is not (Loewith et al., 2002).

**FIGURE 1:**
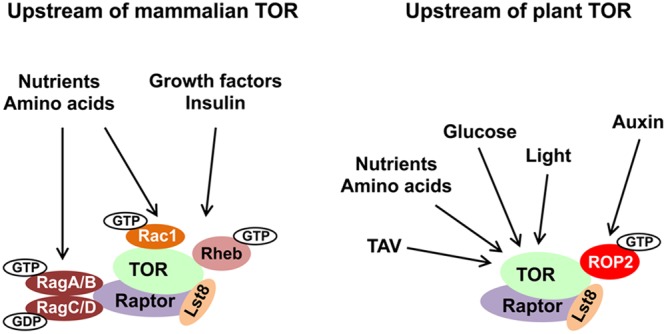
Evolutionary conservation of upstream TOR signaling pathway in plants and animals. The main inputs upstream of TOR are depicted. TOR, Raptor and Lst8 form the core of the TORC1 complex and are conserved in all eukaryotes. Small GTPases represent the best described regulators of TOR kinase. See text for details and abbreviations.

Plants depend greatly on signal perception by TOR (the *Arabidopsis* genome contains a single essential *TOR* gene; Menand et al., 2002; Deprost et al., 2007), which is required to adapt growth and development rapidly to changes in environmental inputs (**Figure 1**). The TOR pathway is a major growth regulator in plants. Previous research with transgenic *Arabidopsi*s plants characterized by increased or decreased *TOR* cellular levels (Deprost et al., 2007) revealed a correlation between both root and shoot growth and *TOR* expression levels, thus confirming a role of TOR in growth regulation. Mutations in the *TOR* gene is lethal, and cause an early block in embryo development (Menand et al., 2002; Deprost et al., 2005). The *Arabidopsis* genome encodes two copies each of *Raptor* and *Lst8* genes. The *Arabidopsis* ortholog of Raptor contains HEAT repeats and WD40 domains responsible for protein–protein interactions, and serves as a binding partner of TOR in complex assembly (Anderson et al., 2005; Deprost et al., 2005). Lst8 consists of seven WD40 repeats, which form a propeller-like structure. Disruption of *Lst8* results in growth retardation phenotypes and extreme sensitivity to shifts in light conditions (Moreau et al., 2012). Recent data suggest that TOR signaling also affects cell wall biogenesis (Leiber et al., 2010) and negatively regulates autophagy in plants (Liu and Bassham, 2010; Zvereva et al., 2016)—a protein degradation process by which cells recycle cytoplasmic content under stress conditions or during senescence. The great enigma of plant TOR biology is the existence of a TORC2 complex, since no homologs of Rictor and Sin1 have been found in the genomes mono- or dicotyledonous plants to date. A search for TOR complex subunit paralogs revealed broad conservation, with a surprising lack of TORC2 in plants and some parasites (Dam et al., 2011; Dobrenel et al., 2016). Unlike TORC2, TORC1 shows a high degree of functional conservation in both multicellular plants and unicellular algae, as manifested by TOR protein–protein interaction experiments (Mahfouz et al., 2006; Díaz-Troya et al., 2008; Moreau et al., 2012).

In mammals, hormones and growth factors can directly activate mTOR signaling via phosphorylation of membrane-bound receptor kinases. Binding of insulin—a major energy control hormone—to receptor tyrosine kinase (RTK) triggers recruitment and phosphorylation of insulin receptor substrate (IRS) adaptors. IRSs activate phosphatidylinositol-3-kinase (PI3K) to generate phosphoinositol (3,4,5)-triphosphate (PIP_3_) (Burke and Williams, 2015). PIP_3_ binds plekstrin homology (PH) domain and mediates the phosphoinositide-dependent kinase 1 (PDK1) and AKT kinase recruitment to the PIP_3_-containing compartments in the PM (Pearce et al., 2010). PDK1-activated AKT phosphorylates TSC2 to inhibit the TSC complex by inducing its release from the lysosome (Alessi et al., 1997). The TSC complex functions as a GAP for Ras homolog enriched in brain (Rheb) small GTPase (Inoki et al., 2003). Rheb is located within the lysosomal compartment, where GTP-loaded Rheb activates mTORC1 via direct interaction with the catalytic domain of mTOR (Long et al., 2005). Availability of nutrients, in particularly amino acids, promotes mTORC1 activity via the conserved Rag family of small GTPases (González and Hall, 2017).

Due to their autotrophic lifestyle, plants lack several key upstream effectors of the TOR complex (e.g., TSC, AKT, and several classes of PI3K). In plants, the most critical environmental input comes from light energy, and suppression of TOR activity negatively affects light-energy-dependent growth (Ren et al., 2012). Upon nutrient deprivation conditions, TOR activity in plants is modulated via potential antagonistic crosstalk with SnRK1 kinase (sensor of cellular energy homeostasis) (Nukarinen et al., 2016); light and sugar signaling through TOR maintain the balance between hormone-promoted growth and carbon availability (Xiong et al., 2013; Dong et al., 2015). Active TOR promotes accumulation of the brassinosteroid-signaling transcription factor BZR1 in response to environmental signals and hormones (Zhang et al., 2016). Thus, TOR kinase represents an evolutionary conserved regulator of metabolism. In plants, disruption of the TOR signaling pathway affects sugar metabolism (Dobrenel et al., 2013). TOR senses and transduces photosynthesis-derived signals to specifically control root meristem proliferation. Glucose promotes primary root and root hair growth via the TOR pathway (Xiong and Sheen, 2012). Glucose-TOR signaling was implicated in transcriptional control of the cell cycle (Xiong et al., 2013).

Another integral part of the mammalian machinery that stimulates mTOR is phospholipase D (PLD) (Wiczer and Thomas, 2012). PLD enzymes harbor a phospholipid-binding Pox domain (PX) and catalyze the hydrolysis of phosphatidylcholine to phosphatidic acid (PA). PA is a metabolite and secondary lipid messenger, which regulates response to growth factors, stress and nutrients. In response to nutrients, PI3K generates PI3P species, which interact with the PX domain of PLD and promote production of PA. PA binds the FRB (FKBP12-rapamycin binding) regulatory domain of mTOR and displaces the DEPTOR subunit from mTOR to rapidly activate mTORC1 (Fang et al., 2001; Yoon et al., 2015). In plants, PLD mediates stress responses and signal transduction. Plant TOR can be a key potential target of PA messengers produced by PLD. Changes in lipid composition and membrane integrity upon various abiotic stresses provoke PLD activity (Bargmann and Munnik, 2006). PA-mediated stomatal closure, root growth, tolerance to salinity and water deficits are the subjects of intensive research in plant science. In plants, PLD is induced by the stress hormone abscisic acid (Jacob et al., 1999). The involvement of PLD in ABA responses raises intriguing questions as to the potential role of abiotic stress and abscisic acid in TOR activation. Moreover, the TOR signaling pathway is involved in the regulation of ABA levels in *Arabidopsis* (Kravchenko et al., 2015). Strikingly, PLD and PA are required for auxin responses, providing hints of crosstalk between auxin and phosphatidylinositol signaling pathways (Li and Xue, 2007). There is now a growing body of evidence demonstrating that TOR acts as an essential factor for auxin signal transduction in *Arabidopsis* (Schepetilnikov et al., 2013; Dong et al., 2015; Deng et al., 2016). Auxin has been also identified as the cellular candidate for a role as an upstream TOR effector (Schepetilnikov et al., 2013). In response to auxin signaling, the TOR pathway is activated as manifested by phosphorylation of the 40S ribosomal S6 kinase 1 (S6K1; a direct downstream target of TOR) at TOR-responsive Thr449, and association of active TOR with polyribosomes. Recently, glucose and light signals as well as exogenously applied auxin were shown to activate S6K1, in shoot meristems (Li et al., 2017).

Phosphorylation is a common post-translational modification that indicates an active status of mTOR kinase. Only three phospho-sites have been reported to date in mTOR (Ser 1261, 2448, and 2481; Acosta-Jaquez et al., 2009). A Rheb-driven phosphorylation event at mTOR Ser1261 within the HEAT repeat domain promotes autokinase activity at Ser2481, resulting in mTOR activation, while the C-terminal Ser2448 is likely phosphorylated by S6K1 via a feedback loop. Mapping of orthologous phosphorylation sites in *Arabidopsis* reveals the high conservation of mammalian Ser2448 and plant Ser2424 epitopes (Schepetilnikov et al., 2013). To date, Ser2424 is the only the TOR specific phospho-site with a confirmed biological function in auxin and ROP2 signaling (Schepetilnikov et al., 2013, 2017).

Many animal viruses have developed multiple mechanisms to activate mTOR signaling in favor of viral replication cycles. One such strategy results in stimulation of the PI3K-AKT pathway upstream of TOR kinase (for a review, see Walsh et al., 2013). The plant pararetrovirus, *Cauliflower mosaic virus* (CaMV), appears to be the first among plant and mammalian viruses known to trigger TOR activation (Schepetilnikov et al., 2011). Indeed, viral transactivator/viroplasmin (TAV) protein binds TOR directly, triggering its activation and recruitment to polysomes. TAV represents a unique example of a pathogenicity effector that specifically targets a basal defense system of plants and suppresses innate immune responses to non-viral pathogens in a TOR-dependent manner (Zvereva et al., 2016).

## Small Gtpases Control the Function and Localization of TOR Complexes

The molecular mechanism of TOR activation is complex and diverse. Small GTPases emerge as the most significant direct upstream regulators of TOR complexes, and function as molecular switches, which, upon activation, interact with downstream effectors and stimulate multiple signaling pathways (**Table 1**). It is well established that yeast and mTOR are regulated by a plethora of small GTPases, including Rho, Rheb, Rag, Rac, Ral, Arf, and Rab, each responsible for perception of a unique type of stimulus. In mammals, small GTPases from the Rheb and Rag families are the two main direct upstream regulators of TOR complexes. Mammalian TORC1 is controlled primarily by Rheb GTPase. However, activation of mTORC1 in response to amino acids requires GTPases of the Rag family (Sancak et al., 2008). Two heterodimeric Rag complexes (RagA/C and RagB/D) bind lysosomal membranes via a lysosomal adaptor RAGULATOR—a scaffold complex with GEF activity toward Rag GTPases (Bar-Peled et al., 2012). Amino acids promote the reciprocal charging of RagA/C and RagB/D with GTP and GDP, respectively, and their binding to mTORC1 via Raptor to relocate mTORC1 to lysosomes for mTORC1 presentation to GTP-bound Rheb (Kim et al., 2008; Sancak et al., 2008). Interestingly, Rac1, a member of the Rho family of small GTPases, affects signaling through both mTORC1 and mTORC2 complexes. Rac1 regulates TOR intracellular localization: upon serum stimulation, Rac1 binds mTOR directly via its C-terminal, lysine-rich motif in a GTP-independent manner and governs its movement from the perinuclear region to the PM (Saci et al., 2011). Thus, Rac1-mediated mTOR activation is independent of the PI3K-AKT-TSC axis. This is opposite to Rheb GTPase, which must be in the GTP-bound state to activate mTOR.

**Table 1:** Small GTPase regulators of TOR complexes.

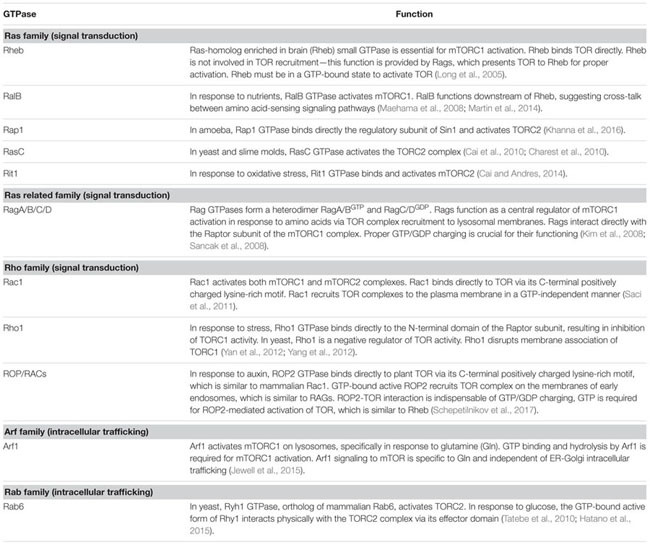

Several amino acids can stimulate mTORC1 in a Rag GTPase independent manner. Glutamine-mediated mTORC1 recruitment to lysosomes requires an alternative pathway via the Arf family GTPase Arf1, which is normally involved in intracellular vesicular trafficking, and vacuolar ATPase (v-ATPase) (Jewell et al., 2015). Several other small GTPases have been identified as indirect upstream actors in the TOR signaling pathway. In yeast, glucose activates TORC2 via the Rab family GTPase Ryh1 (Tatebe et al., 2010; Hatano et al., 2015). Moreover, a member of the Rho GTPase family, Rit, was suggested to bind directly to the hSin1 subunit and activate the mTORC2 complex in response to oxidative stress (Cai and Andres, 2014). Recent data suggest a cross-talk between GTPases RalB and Rheb in nutrient perception and mTORC1 control (Maehama et al., 2008; Martin et al., 2014). Among many small GTPases in yeast, Rho1 GTPase of the Rho family can negatively regulate mTORC1 under stress conditions. Rho1 GTPase is the master regulator of the yeast cell wall integrity (CWI) pathway that controls actin polarization, cell morphogenesis, and cell wall expansion (Levin, 2005). In response to environmental or intracellular stresses, Rho1 binds directly to the Raptor subunit and inhibits mTORC1 activity (Yan et al., 2012; Yang et al., 2012).

In eukaryotes, small regulatory G domain proteins of Ras superfamily are divided into five main families based on their structure, sequence and function: Ran GTPases function in nuclear trafficking; Rab and Arf/Sar—in intracellular vesicular trafficking; Ras and Rho family members regulate signal transduction. ROPs of plants are structurally distinct from the proteins in the Rho, Rac, and Cdc42 subfamilies of Rho GTPases of other eukaryotes (Brembu et al., 2006), but were originally defined as RACs based on sequence similarity to animal Rac GTPases. Since plants lack orthologs of Rheb and Rag GTPases, the ROP/RACs are the only candidates for plant-specific TORC1 upstream regulators. Strikingly, ROP2 interacts directly with TOR both *in vivo* and *in vitro* in a manner independent of its GTP-bound state, but it activates TOR, when bound to GTP. Accordingly, *Arabidopsis* plants with high endogenous auxin levels, or *Arabidopsis* seedlings treated by auxin, or expressing high GTP-bound ROP2 levels, are characterized by increased TOR phosphorylation (Schepetilnikov et al., 2013, 2017). As expected, TOR phosphorylation in response to auxin is abolished in *rop2 rop6 ROP4 RNAi* plants (Schepetilnikov et al., 2017). As expected, *Arabidopsis* plants expressing constitutively active GTP-bound ROP2 (*CA-ROP2* line) are more resistant to TOR inhibitors, and display a significant delay in AZD-8055-sensitive suppression of primary root growth and root hair elongation (Schepetilnikov et al., 2017), as normally occurs in WT seedlings in response to this TOR inhibitor (Montané and Menand, 2013). Similarly, active GTP-bound ROP2 triggered root hair elongation, but, in addition, ROP2 up-regulation promotes initiation of additional misplaced hairs (Jones et al., 2002).

In plants, as with most small GTPases, membrane association of ROPs is mediated by post-translational modifications, including prenylation and *S*-acylation (Hancock et al., 1989; Li et al., 1999; Fu et al., 2005, 2009; Sorek et al., 2011), similar to that shown for members of the Ras superfamily of small G-proteins (Michaelson et al., 2001). Recent research has revealed that ROPs 1–6 and mammalian Rac1 share a common sequence motif comprising several basic lysine residues that direct interaction with TOR (Saci et al., 2011; Schepetilnikov et al., 2017). The next important issue to be resolved is the intracellular compartmentalization of TOR upon auxin treatment. The PM operates as a platform for diverse receptor signaling and vesicle trafficking events. Active GTP-bound ROPs associate closely with the PM, which allows recruitment of ROPs from the cytoplasm (Sorek et al., 2011). ROP GTPases are not known to be localized in intracellular vesicles, suggesting rather a transient association with intracellular compartments or a unique redistribution in the PM. Since ROP2 interacts physically and functionally with TOR, it may participate in TOR relocation to the PM. Interestingly, ROP2 association with PM is indispensable for subsequent TOR activation—ROP2 GTPase lacking a prenylation domain is still capable of interacting with, but not activating, TOR (Schepetilnikov et al., 2017). Phosphorylated TOR accumulates in microsomal fractions of CA-ROP2 plants, and colocalizes with endosomes in the cytoplasm in a ROP2-dependent manner. Note that the Lst8 subunit has been also found colocalized with endosomes (Moreau et al., 2012). TOR binding to endosomes is not sensitive to disruption of ER-to-Golgi intracellular vesicular trafficking, but may rely on the endocytic pathway. Primarily, ROP GTPases are considered to control cytoskeleton reorganization, thus interfering with vesicular trafficking. In response to auxin, GTPases of the ROP family coordinate the recycling of PINFORMED (PIN) transporters between the PM and endomembrane compartments (Chen and Friml, 2014). Accordingly, TOR may move to specific intracellular locations via interaction with appropriate subsets of small regulatory GTPases.

Many questions remain unanswered: what are the effects of ROP2 that increase intrinsic phosphorylation activity of TOR, and do other ROPs contribute to TOR activation? TOR complexes have been found at several subcellular locations, including the cytoplasm and the nucleus. Nevertheless, how TOR can mediate activation on lysosomes and be translocated to 40S preinitiation complexes (40S PIC) to regulate the cell translation machinery is still an open question. In addition, TOR is known to be localized in mitochondria, the PM and stress granules in response to different inputs (Betz and Hall, 2013). In the unicellular green alga *Chlamydomonas*, TOR activity is restricted to ER membranes (Díaz-Troya et al., 2008, 2011). Further work is obviously required to examine the intracellular location and trafficking of TOR, in both active and inactive states, and whether TOR activation takes place before or after its loading on endosomes.

## TOR Promotes Translation Reinitiation in Plants

Plants are sessile organisms that continuously monitor and transduce environmental inputs into regulation of protein synthesis pathways. Indeed, much effort has been directed to demonstrate that translation of many mRNAs is affected by a multitude of environmental signals, for example, cold (Juntawong et al., 2013), heat (Matsuura et al., 2013), dehydration (Kawaguchi et al., 2004; Kawaguchi and Bailey-Serres, 2005; Park et al., 2012), salinity (Park et al., 2012), hypoxia (Branco-Price et al., 2005, 2008), and light (Khandal et al., 2009; Juntawong and Bailey-Serres, 2012; Floris et al., 2013). However, the underlying molecular mechanisms that affect protein synthesis efficiency are largely unknown and in need of further research. A recent study revealed that heat stress can rapidly induce an mRNA degradation process where involving LARPs (La and related Proteins) (Deragon and Bousquet-Antonelli, 2015). Strikingly, mammalian LARP1 was implicated in translation regulation of TOP (5′-terminal oligopyrimidine tract)-containing mRNAs under the control of TOR (Tcherkezian et al., 2014); however, whether translation of many plant TOP-containing mRNAs (Dobrenel et al., 2016) depends on TOR remains to be identified. Moreover, the contribution of TOR to the overall control of cap-dependent translation initiation via phosphorylation of eIF4E-binding proteins (4E-BPs)—the best studied mechanism of translation control in response to stress in other eukaryotes (Siddiqui and Sonenberg, 2015)—has been questioned in plants due to the lack of data on plant 4E-BPs. A discussion of cap-dependent translation control in plants, including a key mechanism of down-regulation of translation by phosphorylation of eIF2α, is beyond the scope of this review, and it is well described recently (Browning and Bailey-Serres, 2015).

**FIGURE 2:**
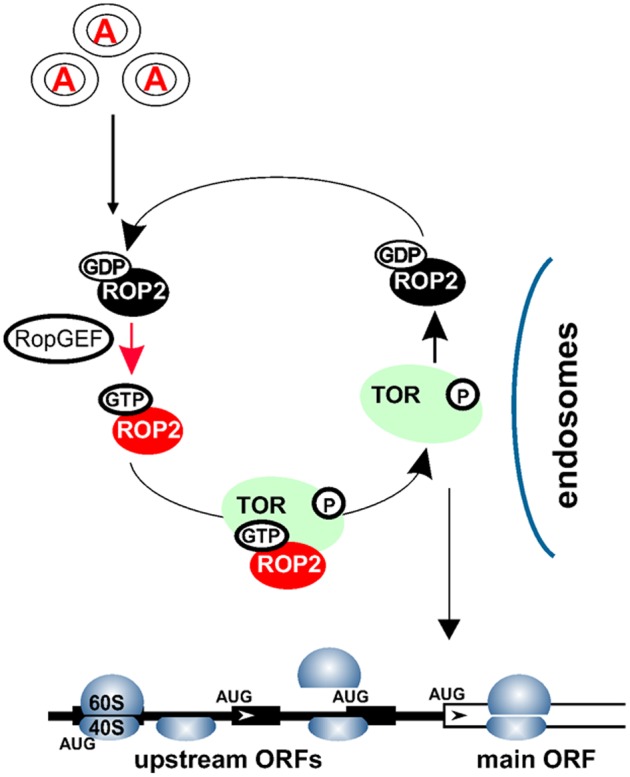
Auxin signaling pathways within the cytoplasm. Auxin signal is recognized via an as yet uncharacterized receptor(s) in the target cells, and transmitted to the cytosol. Auxin mediates recycling of small GTPases—ROP2-GDP to ROP2-GTP—by several GEFs. ROP2 interacts directly with TOR and activates TOR kinase if ROP2 is bound to GTP. TOR activation could occur upon complex formation with ROP2 on earlier endosomes. ROP2 then dissociates from TOR and requires recycling. Active TOR is targeted to eIF3-containing preinitiation complexes and polysomes, where it promotes translation reinitiation of uORF-mRNAs.

Conversely, *Arabidopsis* plants silenced for TOR expression display significantly reduced polysomal abundance (Deprost et al., 2007), suggesting a role for TOR in plant translation. Additionally, it was reported that auxin signaling can affect translation, as manifested by phosphorylation of ribosomal protein S6 (RPS6) and up-regulation of polysomal levels in *Arabidopsis* suspension cultures (Beltrán-Peña et al., 2002; Turck et al., 2004). Accordingly, application of new generation TOR inhibitors, as well as existing TOR-deficient plants, has uncovered TOR function in the translation reinitiation of a specific pool of cellular mRNAs that harbor upstream open reading frames (uORFs) within their leader regions (uORF-mRNAs; Schepetilnikov et al., 2013). The current model suggests that TOR can receive signals from auxin via a small GTPase ROP2 to boost production of important regulatory proteins in a post-transcriptional manner by targeting a specific translation mechanism: reinitiation (**Figure 2**).

Upstream open reading frames are defined as 5′-UTR *cis*-elements of mRNAs defined by a start codon that is out-of-frame with the main ORF. Mounting data suggest the critical importance of post-transcriptional control via translation reinitiation (Roy et al., 2010; Schepetilnikov et al., 2013; von Arnim et al., 2014). Nowadays, uORFs are considered as prevalent translation repressors in eukaryotes (Johnstone et al., 2016). This is not surprising since more than 30% of eukaryotic mRNAs harbor relatively long leaders that contain multiple uORFs (Calvo et al., 2009). Among these are ARF family of transcription factors (von Arnim et al., 2014) and human tyrosine kinases (Wethmar et al., 2016); uORFs play a role of molecular switches in pathophysiology (Wethmar et al., 2010) and in stem cell regulation and organogenesis in plants (Zhou et al., 2014). To understand how translation reinitiation is controlled by upstream signals and contributes to overall protein synthesis, we first review briefly how uORFs can alter expression of the main ORF located downstream of the leader. The scanning model of eukaryotic translation initiation states that the 40S ribosomal subunit prebound by a multisubunit complex (eIF3, eIF1 and eIF1A, eIF5) and a ternary complex (TC, eIF2-GTP-Met-tRNAi^Met^) loads at the capped 5′-end of mRNA via eIF4F-bound to cap, scans in a 3′-direction until it recognizes an initiation codon in a suitable initiation context, where 60S joins and translation elongation begins (Browning and Bailey-Serres, 2015; Hinnebusch et al., 2016). The preceding translation event would negatively interfere with translation reinitiation at a downstream ORF, mainly due to loss of eIFs that have been recruited during the cap-dependent initiation event. It is generally accepted that reinitiation at the downstream AUG codon can occur, if (1) the initiation context of the 5′ AUG codon is not optimal and is recognized only inefficiently by scanning ribosomes (leaky scanning mechanism—Kozak, 1986), and there is no downstream secondary structure that would improve its recognition (Kozak, 1990); or (2) the initiation context is optimal, but is located in close proximity to the 5′-end of mRNA (Kozak, 1991); and (3) the preceding translation event was short (short uORF of 2 to ∼30 codons) (Kozak, 1999). In the latter case, reinitiation is less efficient, but can be improved slightly by having a sufficiently long intercistronic distance between the uORF and the “main” ORF (Kozak, 1987; Luukkonen et al., 1995; Hinnebusch, 1997). The reinitiation potential of ribosomes depends on specific features of uORFs, as well as their amount and combination (von Arnim et al., 2014), and can be regulated by specific *trans*-acting factors (Rahmani et al., 2009; Medenbach et al., 2011).

Beside these features of uORFs, stalled translation of sequence-specific short uORFs can block translation reinitiation of a leader downstream ORF (Sachs and Geballe, 2006). Sequence-specific uORFs are common in genes involved in a variety of control mechanisms, and encode attenuator peptides that act in a sequence-dependent manner to inhibit its own translation termination, often through a delay of peptidyl-tRNA hydrolysis in response to saturating levels of a regulatory signal, usually a metabolite. For example, a 48–55 codon uORF is responsible for the translational repression of the *SAMDC* (*S-ADENOSYLMETHIONINE DECARBOXYLASE*) gene in response to stress conditions and high polyamine levels (Hanfrey et al., 2005). Sequence-specific uORFs control the synthesis of AtbZIP transcription factors and several of their paralogs, as well as trehalose-6-phosphate phosphatase, in a manner sensitive to carbohydrates (Wiese et al., 2004; Hayden and Jorgensen, 2007). It seems certain that more short sequence-specific uORFs that would irreversibly abolish reinitiation of the main ORF translation in response to various regulatory signals or under certain conditions will be identified.

How does the 40S terminating subunit solve the problem of rapid loading of factors necessary for the reinitiation event, i.e., Met-tRNAi^Met^ and 60S? The most likely explanation is that initiation factors that have been recruited during the cap-dependent initiation event dissociate from 40S gradually, and might remain associated with the translating ribosome for a few elongation cycles (Kozak, 1987). These reinitiation promoting factors (RPFs) could assist 40S ribosomal subunits to resume scanning, rapidly acquire TC and 60S *de novo* and thus stay reinitiation-competent. A new study using *in vivo* RNA-protein Ni^2+^-pull down assay directly demonstrated that eukaryotic initiation factor 3 (eIF3) physically associates with early elongating ribosomes on the GCN4 mRNA (Mohammad et al., 2017). eIF3 is composed of 13 distinct subunits in humans and plants, and facilitates rapid recruitment of TC to 40S and assembly of the 43S preinitiation complex on mRNA (Burks et al., 2001; Hinnebusch, 2006). Mammalian RPFs include, in addition to eIF3 (Cuchalová et al., 2010; Munzarová et al., 2011), the cap-binding complex eIF4F (Pöyry et al., 2004).

In plants, eIF3 non-core subunit h (eIF3h) greatly elevates the reinitiation competence of mRNAs coding for the *Arabidopsis* basic zipper transcription factors (bZIPs) and several ARFs (Roy et al., 2010; Zhou et al., 2010); while the 60S ribosomal protein L24B (RPL24B), which is encoded by SHORT VALVE1, lifts translation of uORF-containing mRNAs that encode ARF3 (ETTIN) and ARF5 (MONOPTEROS; Nishimura et al., 2005; Zhou et al., 2010). Thanks to a mutant allele of *eif3h-1* carrying a C-terminally truncated eIF3h and a short valve1 (*stv1*) mutant lacking the RPL24-encoding gene, it was demonstrated that both mutants display similar defects in auxin-mediated organogenesis and undertranslate uORF-containing *bZip11* and several *ARF* mRNAs (Zhou et al., 2010). Although eIF3h can be dispensable for cap-dependent translation initiation (Kim et al., 2004; Roy et al., 2010), a global analysis of ribosomal loading confirmed that many mRNAs containing uORFs are less abundant in polysomes in the *eif3h-1* mutant (Tiruneh et al., 2013), thus confirming that translation of the majority of uORF-containing mRNAs depends on eIF3h. Future studies will clarify the mechanism of eIF3h function in reinitiation of translation.

Taken together, translation of mRNAs with several short uORFs is still possible, albeit with lowered efficiency, while reinitiation after long ORF translation is largely prohibited in eukaryotes. However, viruses often break basic cellular rules. Indeed, there are a few abnormal cases of reinitiation after long ORF translation, best studied in mammalian caliciviruses (Royall and Locker, 2016) and plant caulimoviruses (Ryabova et al., 2006). The subgenomic mRNA of caliciviruses is bicistronic, with two long ORFs that encode structural proteins VP1 and VP2 overlapping by four nucleotides, and its translation relies on a termination-dependent reinitiation strategy, where expression of the downstream cistron is dependent on the ribosome binding site (TURBS) within the upstream VP2 ORF located close to VP1 ORF stop codon. The motif was shown to bind 40S ribosomal subunits and eIF3 (Luttermann and Meyers, 2007; Pöyry et al., 2007). Thus, in caliciviruses, the ribosome might be held at the stop/restart region by base pairing of TURBS with the 18S rRNA (Luttermann and Meyers, 2009; Zinoviev et al., 2015), and can be further stabilized by binding of eIF3 to promote reinitiation by post-terminating 80S ribosomes (Pöyry et al., 2007).

The second unique example of reinitiation after long ORF translation comes from CaMV, where reinitiation critically depends on a single viral protein TAV (De Tapia et al., 1993). To promote reinitiation, TAV interacts with the host translation machinery via eIF3 (Park et al., 2001), reinitiation supporting protein (RISP; Thiébeauld et al., 2009), and TOR, where TAV activates TOR via an as yet unknown mechanism (Schepetilnikov et al., 2011). According to the current model, TAV is responsible for retention of RPFs on translating ribosomes during the long elongation event, thus increasing the reinitiation competence of ribosomes. Indeed, sucrose gradient analysis of extracts isolated from *Arabidopsis* plants transgenic for TAV revealed greatly increased accumulation of eIF3, RISP, and TOR in addition to TAV in polysomes as compared with WT plants (Thiébeauld et al., 2009). Moreover, TAV function in reinitiation is strongly dependent on active TOR (Schepetilnikov et al., 2011). RISP appears to be a specific target of TOR/S6K1 signaling, and its phosphorylation promotes both its binding to TAV and TAV function in translation reinitiation. Indeed, TOR and RISP binding to polyribosomes correlates with RISP phosphorylation, while phosphorylation of RISP is abolished in polysomes isolated from plants transgenic for a TAV deletion mutant that failed to associate and thus activate TOR (Schepetilnikov et al., 2011). In conclusion, it was proposed that TOR function in polysomes would be to maintain the high phosphorylation status of RISP, and possibly other RPFs, to promote viral pathogenesis.

In mammals, eIF3 was identified as a platform for phosphorylation of S6K1 by TOR, where active mTOR or inactive mS6K1 enter the cell translation machinery via interaction with the eIF3-containing preinitiation complex in a dynamic order of events (Holz et al., 2005). Although the eIF3 complex is prebound by inactive mS6K1 in mTOR inactivation conditions, binding of mTOR, when it is activated, results in phosphorylation and dissociation of mS6K1. In yeast, TORC2 was detected in polysomes, where it maintains co-translational phosphorylation of Akt kinase (Oh et al., 2010). In *Arabidopsis*, TOR, when active, associates with polysomes also prebound by inactive S6K1, phosphorylates S6K1, triggering its dissociation (Schepetilnikov et al., 2011, 2013). Further phosphorylation of S6K1 may involve PDK1 (Deak et al., 1999).

In plants, active TOR accumulates mainly within 40S preinitiation complexes, and at significantly lowered levels in polysomes, which can explain the low reinitiation capacity of *Arabidopsis* plants. Although partial depletion of TOR revealed defects in polysomal loading of uORF-containing mRNAs that require reinitiation; TOR, when up-regulated in response to either auxin or by GTP-ROP2, promotes polysomal loading and translation of *ARF3, ARF5, bZIP11*, and other uORF-containing mRNAs (Schepetilnikov et al., 2013, 2017). Active TOR can up-regulate translation reinitiation via phosphorylation of the plant reinitiation factor eIF3h in polysomes to maintain the high phosphorylation status of eIF3h-promoting reinitiation events (Schepetilnikov et al., 2013). A new study has identified mTORC1 as the key factor contributing to translation of uORF-mRNA that encodes ATF4, a member of the CREB/ATF family of bZIP transcription factors, where TOR may regulate ATF4 mRNA translation through a uORF-dependent mechanism and 4E-BPs (Park et al., 2017).

Translation/reinitiation events within *bZIP11, ARF3*, and *ARF5* 5′-UTRs impede or block ribosomal movement toward the main ORF, causing inefficient translation of uORF-mRNAs under WT conditions (Zhou et al., 2010). Indeed, it was shown that uORFs downregulate main ORF translation for *ARF5* by 15-fold, *ARF3* by 2-fold (Zhou et al., 2010), and *bZIP11* by 4-fold, if only uORFs 1, 2, and 4 are removed (Kim et al., 2004). Accordingly, polysomes isolated from WT *Arabidopsis* are deficient in loading of active TOR and *bZIP11, ARF3* and *ARF5* uORF-mRNAs, and their levels are not much higher than in plants grown on medium containing the TOR inhibitor AZD-8055 (Schepetilnikov et al., 2013, 2017). Here, the classic means of determining whether up-regulation of gene expression at the translational level has occurred on mRNAs via a shift of these mRNAs into the polysomal fraction is not easily applied to mRNAs that carry multiple uORFs within their long leader regions that would require reinitiation events. Indeed, the increased abundance of initiating/reinitiating 40S, and likely uORF-translating 80S, within their long leaders shifts these mRNAs toward 80S or even light polysomal fractions, even when translation of the main ORF is strongly inhibited, depending on the number and arrangement of uORFs (Schepetilnikov et al., 2017). Upon introduction of TOR-activated conditions, TOR phosphorylation, and, consequently, uORF-mRNA loading into polysomes is increased (Schepetilnikov et al., 2017). Strikingly, studies of mRNA abundance across sucrose gradients in WT versus *CA-ROP2* plants (Schepetilnikov et al., 2017) revealed a high proportion of uORF-mRNA (about 64–80%) sedimenting to the top fraction of the gradient in WT conditions, while only 20–25% of uORF-mRNA remained in the top gradient fraction in *CA-ROP2* conditions, regardless of the fact that total transcript levels did not differ significantly between WT and *CA-ROP2* extracts. These data correlate with the high translation efficiencies of uORF-mRNAs in plant mesophyll protoplasts prepared from plants expressing high active TOR levels.

TOR up-regulation of reinitiation events could be as harmful in plants as in mammals, where up-regulation of the protein synthesizing machinery contributes to the development of cancer (Ruggero and Pandolfi, 2003). In the opposite situation of reinitiation defects, the developmental abnormalities identified in rpl24b and *eif3h-1* mutants are largely similar to auxin-related developmental defects (Zhou et al., 2010). Further investigation is needed to understand the roles of ROP2 in TOR activation, as well as to identify other upstream TOR effectors in plants and their roles in translation.

## Conclusion and Perspectives

The last 10 years have witnessed striking advances and rapidly emerging data on the composition of the TOR complex, the TOR pathway, and its function and control in plants, in part due to the appearance of a new generation of TOR inhibitors that bind to the TOR kinase domain within the ATP-binding pocket and inactivate TOR (Chresta et al., 2010; Montané and Menand, 2013). Many critical questions remain unanswered. Recent work has revealed the role of TOR in sensing environmental conditions, including various stresses and phytohormones, but the molecular mechanisms underlying these signaling events remain unknown. It is not yet known whether, and how, TOR controls general translation by sensing amino acid levels. Finally, a key issue is the existence of functional ortholog of TORC2 in plants. Recent data have revealed that the molecular composition of the TOR complex varies in different cell types. Identification of a novel binding partner of TOR—GIT1 (G-protein-coupled receptor kinase-interacting protein 1)—suggested a unique mTOR complex lacking both Raptor and Rictor (Smithson and Gutmann, 2016). Therefore plants can contain more than one functional TOR complex. A challenge for future studies in plants will be to elucidate further TOR signaling pathways in plant translation, and to reveal how TOR can control mRNA translation at the initiation step.

## Author Contributions

All authors listed, have made substantial, direct and intellectual contribution to the work, and approved it for publication.

## Conflict of Interest Statement

The authors declare that the research was conducted in the absence of any commercial or financial relationships that could be construed as a potential conflict of interest.
